# Multiple Drug Treatments That Increase cAMP Signaling Restore Long-Term Memory and Aberrant Signaling in Fragile X Syndrome Models

**DOI:** 10.3389/fnbeh.2016.00136

**Published:** 2016-06-30

**Authors:** Catherine H. Choi, Brian P. Schoenfeld, Aaron J. Bell, Joseph Hinchey, Cory Rosenfelt, Michael J. Gertner, Sean R. Campbell, Danielle Emerson, Paul Hinchey, Maria Kollaros, Neal J. Ferrick, Daniel B. Chambers, Steven Langer, Steven Sust, Aatika Malik, Allison M. Terlizzi, David A. Liebelt, David Ferreiro, Ali Sharma, Eric Koenigsberg, Richard J. Choi, Natalia Louneva, Steven E. Arnold, Robert E. Featherstone, Steven J. Siegel, R. Suzanne Zukin, Thomas V. McDonald, Francois V. Bolduc, Thomas A. Jongens, Sean M. J. McBride

**Affiliations:** ^1^McDonald Laboratory, Section of Molecular Cardiology, Departments of Medicine and Molecular Pharmacology, Albert Einstein College of Medicine, Yeshiva UniversityBronx, NY, USA; ^2^Department of Dermatology, Dermatology Clinic, Drexel University College of MedicinePhiladelphia, PA, USA; ^3^Jongens Laboratory, Department of Genetics, University of Pennsylvania School of MedicinePhiladelphia, PA, USA; ^4^Bolduc Laboratory, Department of Pediatrics, Center for Neuroscience, University of AlbertaEdmonton, AB, Canada; ^5^Zukin Laboratory, Dominick P. Purpura Department of Neuroscience, Albert Einstein College of Medicine, Yeshiva UniversityBronx, NY, USA; ^6^Siegel Laboratory, Translational Neuroscience Program, Department of Psychiatry, University of Pennsylvania School of MedicinePhiladelphia, PA, USA; ^7^Arnold Laboratory, Department of Psychiatry, University of Pennsylvania School of MedicinePhiladelphia, PA, USA

**Keywords:** Fragile X, group II mGluR, lithium, tau, GSK-3β, mTOR, PDE-4

## Abstract

Fragile X is the most common monogenic disorder associated with intellectual disability (ID) and autism spectrum disorders (ASD). Additionally, many patients are afflicted with executive dysfunction, ADHD, seizure disorder and sleep disturbances. Fragile X is caused by loss of FMRP expression, which is encoded by the *FMR1* gene. Both the fly and mouse models of fragile X are also based on having no functional protein expression of their respective *FMR1* homologs. The fly model displays well defined cognitive impairments and structural brain defects and the mouse model, although having subtle behavioral defects, has robust electrophysiological phenotypes and provides a tool to do extensive biochemical analysis of select brain regions. Decreased cAMP signaling has been observed in samples from the fly and mouse models of fragile X as well as in samples derived from human patients. Indeed, we have previously demonstrated that strategies that increase cAMP signaling can rescue short term memory in the fly model and restore DHPG induced mGluR mediated long term depression (LTD) in the hippocampus to proper levels in the mouse model (McBride et al., 2005; Choi et al., 2011, 2015). Here, we demonstrate that the same three strategies used previously with the potential to be used clinically, lithium treatment, PDE-4 inhibitor treatment or mGluR antagonist treatment can rescue long term memory in the fly model and alter the cAMP signaling pathway in the hippocampus of the mouse model.

## Introduction

Fragile X syndrome is the most common monogenic cause of both intellectual disability and autism spectrum disorders (Turner et al., 1996; Crawford et al., 2002; Hagerman, 2008). Fragile X is caused by the loss of *FMR1* gene function, which is caused by transcriptional silencing due to CGG repeat-expansion induced hypermethylation in the promoter region, resulting in loss of the gene product, FMRP. The incidence of Fragile X is roughly 1 in 2500 to 1 in 4000 births (Pesso et al., 2000; Crawford et al., 2001, 2002; Toledano-Alhadef et al., 2001; Hagerman, 2008). Symptoms associated with Fragile X almost always include mild to severe developmental intellectual disability and can include attention deficit hyperactivity disorder, seizures, sleep disorders, anxiety and autism. FMRP is known to be enriched both presynaptically and postsynaptically in neurons, and is associated with and regulates a number of mRNAs in response to synaptic activity (Jacquemont et al., 2007; Christie et al., 2009; Darnell et al., 2011).

A *Drosophila* model for Fragile X syndrome, based on the loss of *dfmr1* expression, recapitulates several phenotypes observed in Fragile X including impairments in social interaction and several phases of memory including immediate recall memory, short term memory and long term memory (Zhang et al., 2001; Dockendorff et al., 2002; Morales et al., 2002; McBride et al., 2005; Bolduc et al., 2008, 2010). Previous studies in *Drosophila* have demonstrated that treatment with lithium, metabotropic glutamate receptor (mGluR) antagonists or phosphodiesterase type 4 (PDE-4) inhibitors can rescue several phenotypes displayed by these flies including courtship (social interaction), cognitive defects and a midline crossing defect of the mushroom bodies in the brain (Dockendorff et al., 2002; McBride et al., 2005; Bolduc et al., 2008; Choi et al., 2010, 2015). However, a critical question when thinking about attempting to move these compounds forward clinically is which of these potential treatments have efficacy in rescuing the long-term memory (LTM) impairments. In *Drosophila* and in mammals, there are distinct phases of learning and memory which have been distinguished through genetic and pharmacologic dissection which often involve overlapping but distinct signaling cascades and circuitry (Tully et al., 1994; Yin et al., 1994; Greenspan, 1995; Yin and Tully, 1996; Kane et al., 1997; Joiner and Griffith, 1999; McBride et al., 1999; Zars et al., 2000; Kandel, 2001; Pascual and Preat, 2001; Kelleher et al., 2004; Yu et al., 2004; Margulies et al., 2005; Guven-Ozkan and Davis, 2014). Therefore treatments that rescue learning/immediate recall memory (0–2 min memory) or short term memory (60 min memory) may not rescue long term memory.

**Figure 1:**
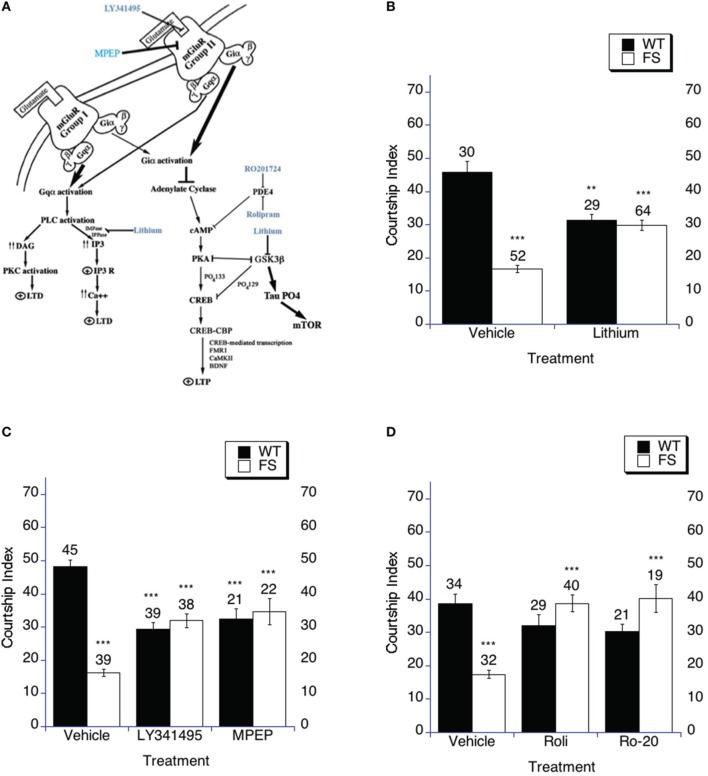
**Targeting the cAMP signaling cascade increases social interaction. (A)** The cAMP signaling cascade. The three treatments used in this study increase cAMP signaling in the fly and mouse. It has been demonstrated that antagonizing or dampening the signaling of either of the mGluR pathways can rescue multiple phenotypes in the fly and mouse models of Fragile X including memory, audiogenic seizure and enhanced mGluR-LTD (McBride et al., 2005; Yan et al., 2005; Dolen et al., 2007; Choi et al., 2010, 2011). Additionally, lithium has demonstrated efficacy in rescuing cognitive abilities, audiogenic seizure and enhanced mGluR-LTD in fly and mouse models as well as in human patients (McBride et al., 2005; Berry-Kravis et al., 2008; Min et al., 2009b; Choi et al., 2010, 2011; Yuskaitis et al., 2010; Liu et al., 2011). As is displayed in the figure, PDE-4 also intersects in this signaling cascade. The common aspect is that lithium, mGluR antagonists and PDE-4 inhibitors should all result in increasing cAMP signaling and increased PKA activity. Each should inhibit GSK-3B activity, which may be read out as phosphorylation of tau. Tau phosphorylation has been demonstrated to phosphorylate and activate mTOR, although many pathways feed into mTOR signaling. **(B–D)** Social interaction (Naive courtship of sham trained males) was examined after drug or vehicle treatment. **(B–D)** Naive courtship of WT and FS flies exposed to drug or drug vehicle control containing food. Mean CIs (±SEM) are plotted; Ns are indicated above each bar for all groups. The comparisons shown on the graphs are between vehicle and drug treatment with the same genotype and between WT and FS treatment for the vehicle treatment. For levels of significance, ^**^p < 0.01; ^***^p < 0.001. Filled bars indicate WT males; open bars indicate FS males. **(B)** Flies were raised on regular food and starting on the 1st day of adulthood placed on either vehicle control food containing 5 mM NaCl vehicle or food supplemented with 5 mM lithium. With vehicle treatment, FS males court virgin females less vigorously than do WT flies (*p* < 0.001) represented by asterisks over the FS vehicle bar. FS flies treated with lithium spend a higher percentage of time engaged in courtship compared to vehicle treated FS flies, asterisks above the FS lithium bar. WT flies treated with lithium demonstrated decreased courtship compared to vehicle treated WT flies, asterisks over the WT lithium bar. **(C)** Flies were raised on regular food and starting on the 1st day of adulthood placed on either vehicle control food containing DMSO vehicle or food supplemented with either 400 nM LY341495 or 8.6 μM MPEP. With vehicle treatment, FS males court virgin females less vigorously than do WT flies, asterisks over the FS vehicle bar. FS flies treated with either MPEP or LY341495 spent a higher percentage of time engaged in courtship compared to vehicle treated FS flies, asterisks over the FS LY341495 and FS MPEP bars. WT flies treated with either MPEP or LY341495 demonstrated decreased courtship compared to vehicle treated flies, asterisks over the WT MPEP and WT LY341495 bars. **(D)** Flies were raised on regular food and starting on the 1st day of adulthood placed on either vehicle control food containing DMSO vehicle or food supplemented with either 50 μM Rolipram (Roli) or 20 μM Ro-20-1724 (Ro-20). With vehicle treatment, FS males court virgin females less vigorously than do WT flies, asterisks over the FS vehicle bar. FS flies treated with either rolipram or Ro-20-1724 spent a higher percentage of time engaged in courtship compared to vehicle treated FS flies, asterisks above the FS rolipram and FS Ro-20-1724 bars. WT flies treated with either rolipram or Ro-20-1724 demonstrated no change in the time spent engaged in courtship compared to vehicle treated flies.

We chose to examine three treatment strategies to examine the efficacy on LTM in the fly model, all of which modulate cAMP signaling in the fly. This is because cAMP signaling has a central role in LTM formation and appears to be suppressed in the fragile X fly and mouse models. Lithium, mGluR antagonists and PDE-4 inhibitors have a common action of up-regulating cAMP signaling. For lithium, the upregulation of cAMP signaling is through the inhibition of glycogen synthase kinase-3beta (GSK-3β) which is a known negative regulator of protein kinase A (PKA; Fang et al., 2000; Li et al., 2000; Tanji et al., 2002; McBride et al., 2005; Walsh et al., 2008; Min et al., 2009a; See Figure 1A). The mGluR antagonists and PDE-4 inhibitors act more directly on the regulation of cAMP levels (See Figure 1A). Adenylate cyclase synthesizes cAMP, which is in turn degraded by phosphodiesterase (PDE) activity, with PDE-4 being the most abundant cAMP specific PDE in the brain of flies and mammals (Davis et al., 1989). PDE-4 inhibitors directly increase cAMP levels after synaptic stimulation, by inhibiting PDE-4 from degrading cAMP. In mammals, Group II mGluRs predominantly couple to Gi signaling and suppress adenylate cyclase activity upon stimulation, therefore antagonists directly prevent the inhibitory actions of Gi signaling on cAMP production and signaling (Sato et al., 2004). Recent studies have also shown that Group I mGluRs can couple to Gi (Kreibich et al., 2004), thus inhibition of both might raise cAMP levels. In *Drosophila*, LY341495 and MPEP both bind DmGluRA, the *Drosophila* mGluR that is coupled to both Gi and Gq signaling. In mammals LY341495 is a Group II mGluR antagonist and MPEP is a Group I mGluR antagonist with the Group I mGluRs being predominantly coupled to Gq signaling.

The most robust endophenotype to date in the Fragile X mouse model is exaggerated metabotropic glutamate receptor (mGluR)-dependent long-term depression (LTD) in the CA1 region of the hippocampus at 6–8 weeks of age to 11 months of age (Huber et al., 2002; Hou et al., 2006; Nosyreva and Huber, 2006; Choi et al., 2011, 2015). It is widely postulated that long-term depression (LTD) as well as long-term potentiation (LTP) may be cellular models of learning and memory. Chronic treatments of fragile X mice with lithium, group I mGluR antagonists, group II mGluR antagonists and PDE-4 inhibitors have been demonstrated to abrogate the enhanced mGluR-LTD phenotype (Choi et al., 2011, 2015; Michalon et al., 2012). In contrast to the *Drosophila* Fragile X model, *Fmr1* KO mice display, at best, subtle behavioral and cognitive deficits that can to be difficult to replicate (Bakker and Oostra, 2003; Spencer et al., 2006). However, several groups have found that audiogenic seizures, open field testing and cerebral protein synthesis phenotypes can be reliably obtained and rescued by lithium or GSK3B inhibition (Min et al., 2009a; Yuskaitis and Jope, 2009; Mines et al., 2010; Yuskaitis et al., 2010; Liu et al., 2011, 2012; Mines and Jope, 2011). More recently, genetic reduction, short-term or long term pharmacologic antagonization of group I mGluRs has successfully rescued some phenotypes associated with the Fragile X mouse (Yan et al., 2005; Hou et al., 2006; Dolen et al., 2007; De Vrij et al., 2008; Michalon et al., 2012). Therefore, herein we complement the fly studies on LTM by also examining the effect of chronic treatments with lithium, group II mGluR antagonists and PDE-4 inhibitors on brain and lymphocyte biochemistry in the Fragile X mouse model in order to get a better understanding of how these findings may translate into clinically beneficial treatments. These treatments are similar to those used in the fly (chronic treatment) and the treatment conditions used are the same as those previously demonstrated to rescue the aberrant mGluR LTD phenotype in the mouse (Choi et al., 2011, 2015).

## Results

### Targeting the cAMP signaling cascade with three distinct pharmacological treatments

We will first describe the treatment paradigms used in our studies then we will describe the results of these studies in the subsequent sections.

#### Using group II mGluR antagonists to up-regulate the cAMP signaling cascade

The mGluRs are G-protein coupled receptors. The *Drosophila* genome contains a single metabotropic receptor (mGluR, DmGluRA), and its characterization indicates it activates signaling pathways downstream of both Gi and Gq (Parmentier et al., 1996; McBride et al., 2005; Pan and Broadie, 2007; Pan et al., 2008; Choi et al., 2010; Kanellopoulos et al., 2012). Gi alpha activation inhibits adenylate cyclase (AC), thereby preventing an increase in cAMP, which prevents PKA (protein kinase A) from phosphorylating the activation site of CREB at serine 133, and thereby reduces CREB-mediated gene transcription, a readout of cAMP mediated signaling relevant to memory. In fact, altering the activity of CREB can enhance memory in *Drosophila* and additionally enhance long-term facilitation in *Aplysia* and LTP in mice (Yin et al., 1994, 1995; Roman and Davis, 2001; Vitolo et al., 2002; Bozon et al., 2003; Chen et al., 2003; Tully et al., 2003; Kanellopoulos et al., 2012). LY341495 is a competitive antagonist of the mammalian group II mGluRs, but has been shown to inhibit the *Drosophila* mGluR, DmGluRA (Bogdanik et al., 2004). MPEP is a selective group I mGluR5 non-competitive antagonist in mammals, which also inhibits DmGluRA in *Drosophila* through a conserved binding pocket for MPEP (McBride et al., 2005). We used both MPEP and LY341495, to inhibit DmGluRA, which in turn will prevent suppression of adenylate cyclase activity maintaining or increasing cAMP signaling in the fly (Kanellopoulos et al., 2012; Choi et al., 2015). DmGluRA is coupled to both Gi and Gq signaling. We used LY341495 in the mouse to antagonize Group II mGluRs again to maintain or increase cAMP signaling in the mouse. We did not use MPEP for mouse work in this study since the focus is on cAMP signaling with regard to Fragile X models (Figure 1A) and in mammals at low doses MPEP is selective for mGluR5 along with having a very short half-life making chronic treatment difficult.

#### Using lithium to up-regulate the cAMP signaling cascade

At therapeutic doses lithium has been shown to inhibit GSK-3β activity (Klein and Melton, 1996), and additionally lithium treatment has been shown to facilitate CREB mediated transcriptional activity (by inhibiting GSK-3B activity) in cultured cells (Bullock and Habener, 1998; Grimes and Jope, 2001; Mai et al., 2002; Son et al., 2003). PKA inhibits GSK-3β activity and GSK-3β has been shown to inhibit PKA activity, therefore inhibiting GSK-3β relieves inhibition from PKA in the cAMP signaling cascade providing the same effect that as a rise in intracellular cAMP which promotes PKA activity (Fang et al., 2000; Li et al., 2000; Tanji et al., 2002; Figure 1A).

#### Using PDE-4 inhibitors to up-regulate the cAMP signaling cascade

Previously studies of cAMP levels have determined that they are low in the brains of both *dfmr1* mutant flies and *Fmr1* knockout (KO) mice (Kelley et al., 2007; Choi et al., 2015) as well as in flies that are heterozygous for a *dfmr1* loss of function mutation (Kanellopoulos et al., 2012). Decreased cAMP levels have also been demonstrated in Fragile X patients (Berry-Kravis and Huttenlocher, 1992; Berry-Kravis et al., 1995; Berry-Kravis and Ciurlionis, 1998). Interestingly, *FMR1* transcription is dependent on CREB mediated transcription in flies and mammals, which is activated by the cAMP pathway (Hwu et al., 1997; Cha-Molstad et al., 2004; Kanellopoulos et al., 2012) and consistent with these findings there is a positive correlation between FMRP levels and cAMP levels in cell lines and platelets derived from afflicted patients (Berry-Kravis and Huttenlocher, 1992; Berry-Kravis et al., 1995; Berry-Kravis and Ciurlionis, 1998; Kelly et al., 2007). This led to the hypothesis that one way to correct some of the phenotypic cognitive deficits in Fragile X syndrome is to increase cAMP to wild type physiologic levels. Although this would not increase FMRP in patients (because it is silenced), it would potentially increase levels of the other effectors in the cAMP signaling cascade, which have been shown to play a role in cognition (Impey et al., 2004). As shown in Figure 1A, PDE-4 inhibition will prevent the breakdown of cAMP by PDE-4 and thereby increase cAMP levels after synaptic stimulation thereby increasing cAMP signaling cascade activity. This strategy has previously been shown to be successful to rescue phenotypes in the mouse and fly models of fragile X (Choi et al., 2015).

Indeed, it has previously been demonstrated that treatment of the Fragile X fly model with lithium, mGluR antagonists or PDE-4 inhibitors could rescue social interaction, immediate recall memory and short term memory in the conditioned courtship paradigm and odor shock paradigm in the fly model (McBride et al., 2005; Bolduc et al., 2008; Choi et al., 2015) and normalize DHPG induced mGluR mediated LTD in the mouse model of fragile X when treatment is started in adulthood (Choi et al., 2011, 2015; Michalon et al., 2012). The three strategies should all increase cAMP signaling and lead to inhibited GSK-3B activity. The decreased GSK-3β activity may be followed by examining one of the well characterized substrates of GSK-3β phosphorylation, which is the microtubule binding protein tau.

### The effects of chronic stimulation of cAMP signaling on social interaction in Fragile X

The *Drosophila* model of Fragile X is based on loss of function of *dfmr1*, the *Drosophila* ortholog of *FMR1*. For our studies, we use a *dfmr1* deletion line carrying a genomic transgene with a frame-shift mutation engineered in the *dfmr1* coding region that is driven by the endogenous promoter, referred to as the FS line. The control line for these studies contains the same deletion of the *dfmr1*gene, but also carries a wild type transgene for *dfmr1* that is driven by the endogenous promoter and is referred to as the WT line (Dockendorff et al., 2002; McBride et al., 2005; Banerjee et al., 2010). Both the FS and WT lines are heterozygous for the transgenes and one copy of the transgene provides approximately the same *dfmr1* protein levels as is seen in control lines. The baseline courtship values in this manuscript are slightly higher than in our previous papers (Dockendorff et al., 2002; McBride et al., 2005) due to a different genetic background as in this paper we use the WT and FS flies in an OreR background for the courtship experiments (Banerjee et al., 2010). The fragile X model flies have previously been shown to display social interaction deficits as assayed by naïve (sham) courtship with virgin female targets (Dockendorff et al., 2002; McBride et al., 2005; Banerjee et al., 2010; Choi et al., 2010). Courtship activity has been studied for over a century in the lab and is an indication of social interaction in *Drosophila* (Sturtevant, 1915; Bastock, 1955, 1956; Spieth, 1974; Hall, 1994). Initially, this was heavily studied in with examining courtship in pairs of males and females and later in pairs of males and immature males. However, although a good approximation of social interaction in reproductive situations, social interaction can now also be studied in assays, which do not intermingle reproductive drive as a variable. The assays eliminating reproductive drive have now become valuable tools in the field (Bolduc et al., 2010; Schneider et al., 2012; McNeil et al., 2015).

Here again we found that although courtship is high enough to expect memory formation after training, the fragile X flies display naïve courtship that is significantly lower than WT (control) flies for all three of the vehicle treatment groups (Figures 1B–D), where the comparison is between the WT vehicle and FS vehicle treatment flies for lithium (Figure 1B), mGluR antagonists (Figure 1C) and PDE-4 inhibitors (Figure 1D). Fragile X flies treated in adulthood with either lithium (Figure 1B), mGluR antagonists (LY341495 or MPEP; Figure 1C) or PDE-4 inhibitors (rolipram or Ro-20-1724; Figure 1D) displayed significantly increased social interaction compared to each group of vehicle treated Fragile X flies, which fits with the previous literature (Figures 1B–D; McBride et al., 2005; Choi et al., 2010, 2015). WT flies treated with either lithium or mGluR antagonists (LY341495 or MPEP) displayed decreased social interaction compared to vehicle treatment, which fits with the previous literature (Figures 1B,C; McBride et al., 2005; Choi et al., 2010). WT flies treated with PDE-4 inhibitors (rolipram or Ro-20-1724) demonstrated a trend toward decreased social interaction, but it did not reach significance (Figure 1D). In sum, these data indicate that increasing cAMP levels in *dfmr1* mutants can rescue social interaction as measured in the courtship assay, but additional increasing of signaling in this pathway is detrimental to normal flies that presumably have optimized levels (see Discussion).

### The effects of chronic stimulation of cAMP signaling on long term memory in Fragile X

#### Lithium treatment in adulthood rescues LTM in Fragile X flies

Herein, we wanted to test if treatments begun in adulthood could rescue LTM, as they have been demonstrated to rescue social interaction and impairments in other phases of memory in the fragile X model. In *Drosophila*, long-term memory can be assessed in several ways and the two we utilize in this paper are the conditioned courtship associative memory paradigm and the olfactory conditioning associative memory paradigm. In the series of experiments conducted during this study we used the conditioned courtship paradigm extensively and we confirmed those findings by doing a subset of key experiments in the olfactory conditioning associative memory paradigm. Each paradigm is able to stand alone as an assay of associative long term memory, but in this case since work on the fragile X fly model had previously been performed in both assays, we decided that it would be helpful to use both assays but in the case of the olfactory paradigm we only used one compound per class of drugs to confirm the findings in the conditioned courtship paradigm. Conditioned courtship and olfactory conditioning (Pavlovian conditioning) have allowed for a genetic dissection of each stage of memory formation from learning to long-term memory in *Drosophila* (Quinn et al., 1974; Siegel and Hall, 1979; Tully and Quinn, 1985; Tully et al., 1994; Yin et al., 1994, 1995; McBride et al., 1999; Banerjee et al., 2010). The conditioned courtship long-term memory paradigm takes advantage of fly courtship behavior. If a male is paired with a previously mated female over the course of 5–7 h (7 h in this paper), his courtship will decrease during the training period due to the female's aversive cues and rejection of his advances. When subsequently paired with a virgin female, 1–9 days later (4 days later is used in this paper), a male will continue to have suppressed courtship relative to a naive/sham trained male that has not been exposed to a previously mated female (McBride et al., 1999). This lower courtship activity is indicative of a memory of the training. The courtship indices (CIs), the percentage of time a male spends courting a female during a timed courtship interval, are used to calculate a memory index (MI; Siegel and Hall, 1979; Keleman et al., 2007; see methods). A larger MI value is indicative of memory and scores closer to zero or negative values are indicative of no memory of the training (Keleman et al., 2007). The fragile X flies have already been shown to have impairments in long-term memory in conditioned courtship (McBride et al., 1999; Banerjee et al., 2010).

Previous studies have identified lithium as a potential treatment for fragile X in fly and mouse models as well as in a pilot trial in patients (McBride et al., 2005; Berry-Kravis et al., 2008; Min et al., 2009a,b; Yuskaitis et al., 2010; Liu et al., 2011, 2012). In mouse studies this has been found, at least in part, to be secondary to the effects of lithium inhibiting GSK-3β activity (Klein and Melton, 1996; Mines et al., 2010, 2013; Yuskaitis et al., 2010). Lithium can inhibit GSK-3β and thereby increase cAMP signaling, whereas cAMP levels may be suppressed by the over-active mGluR signaling in the fragile X fly model, mouse model and cell lines derived from patients (Figure 1A; Berry-Kravis and Huttenlocher, 1992; Berry-Kravis et al., 1995; Berry-Kravis and Ciurlionis, 1998; McBride et al., 2005; Kelley et al., 2007; Choi et al., 2015). Lithium is clinically FDA approved for the treatment of Bipolar Disorder and is used off label for a number of other psychiatric indications.

**Figure 2:**
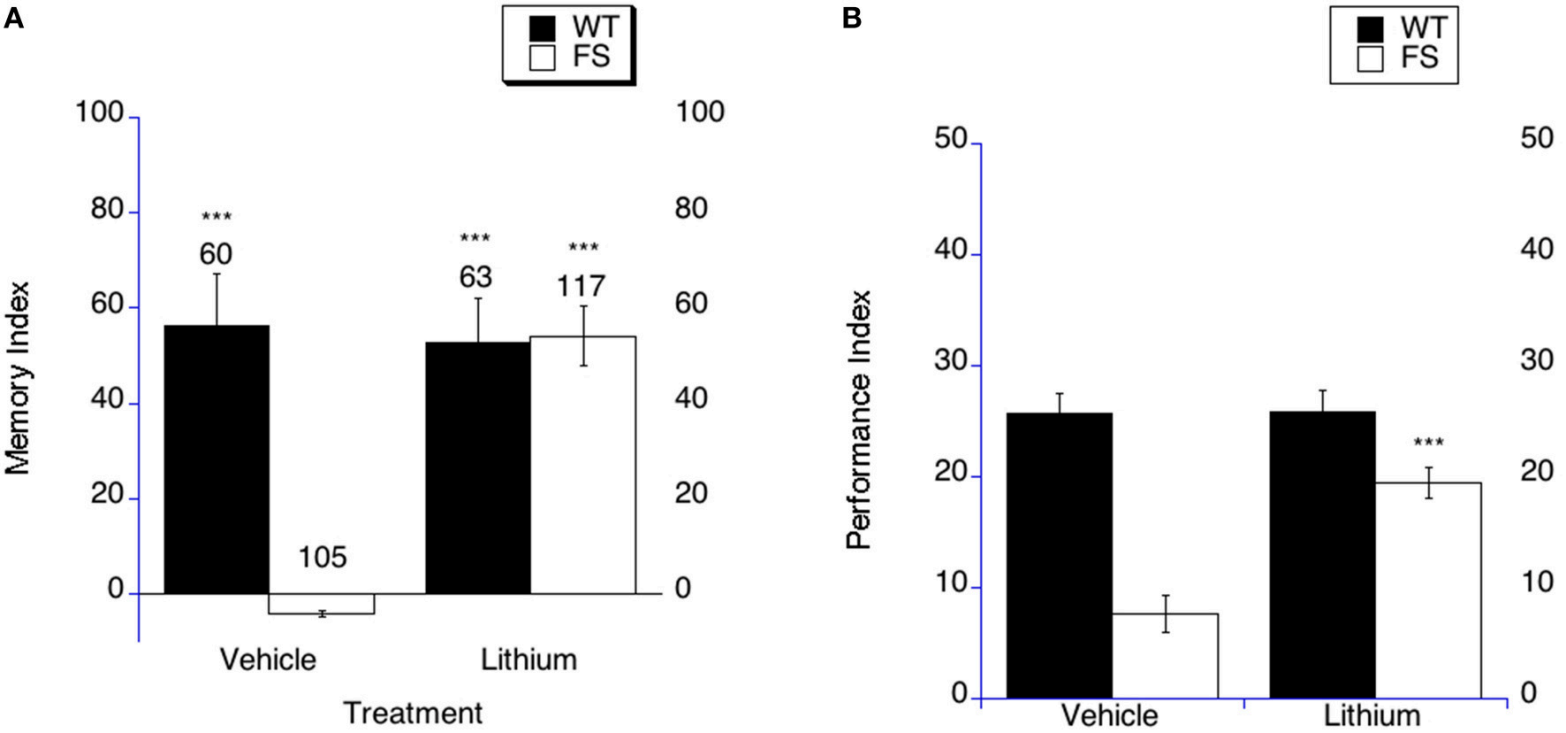
**Long-term memory is rescued by lithium treatment in fragile X flies. (A)** Long-term memory (4 days post training) was measured in WT and FS flies that were administered vehicle control food or drug treatments contained in the food. Memory Index (±the error) was plotted; Ns are indicated above each bar for all groups. The levels of significance are ^*^p < 0.05; ^**^p < 0.01; ^***^p < 0.001, and are generated from comparisons of same genotype, same treatment between LTM trained and sham trained flies. Filled bars indicate WT males; open bars indicate FS males. Flies were raised on regular food and starting on the 1st day of adulthood placed on either vehicle control food containing 5 mM NaCl vehicle or food supplemented with 5 mM lithium. With vehicle treatment, FS males did not display long-term memory. FS flies treated with lithium did demonstrate long-term memory. WT flies treated with either vehicle or lithium demonstrated long-term memory. **(B)** Lithium rescues the olfactory-based long-term memory defect observed in *dfmr1* mutants. Flies were placed on LiCl or vehicle in food for 9 days prior to spaced training. Long-term memory of olfactory conditioning after spaced training was significantly improved in FS mutants after being administered lithium. The levels of significance are indicated as follows: ^***^p < 0.001. No effect of treatment was observed in WT controls.

In order to test the hypothesis that lithium may rescue cognitive impairments in Fragile X flies, FS and WT flies were treated with lithium or the appropriate vehicle (the same concentration of NaCl in the food) for 9 days starting on the day of eclosion as an adult. Flies then underwent sham or LTM training on day 10 and were subsequently placed back on lithium or vehicle containing food for 3 days and tested for memory of the training session on day 4 post training. The results are graphed using a memory index and the results indicate whether or not LTM is present in a particular paired comparison of sham trained compared to LTM trained means. FS flies demonstrated rescued LTM at 4 days post training after treatment with lithium (Figure 2A), meaning there was a suppression of courtship in the LTM trained group compared to the sham trained group when the FS flies were treated with lithium as is graphically shown by the higher memory index (Figure 2A). In contrast, FS flies continued to have impaired LTM when treated with the vehicle control, which fits with previously published literature (Figure 2A). WT flies displayed intact LTM when treated with lithium or vehicle (Figure 2A).

In order to determine if lithium treatment could rescue LTM in the olfactory-based LTM memory paradigm, we examined LTM in FS flies that were treated with lithium or vehicle. It should be noted that in this paradigm the performance indices are compared between different groups. The FS flies on vehicle demonstrate worse LTM compared to the WT flies on vehicle treatment (Figure 2B, *p* < 0.001). FS flies that were treated with lithium displayed improved LTM compared to FS flies on vehicle treatment (Figure 2B as indicated by the asterisks). This represents a performance improvement in the FS flies, but not a full phenotypic reversal. WT flies demonstrated similar LTM on either lithium or vehicle treatment (Figure 2B).

### mGluR antagonist treatment in adulthood rescues LTM in Fragile X flies

**Figure 3:**
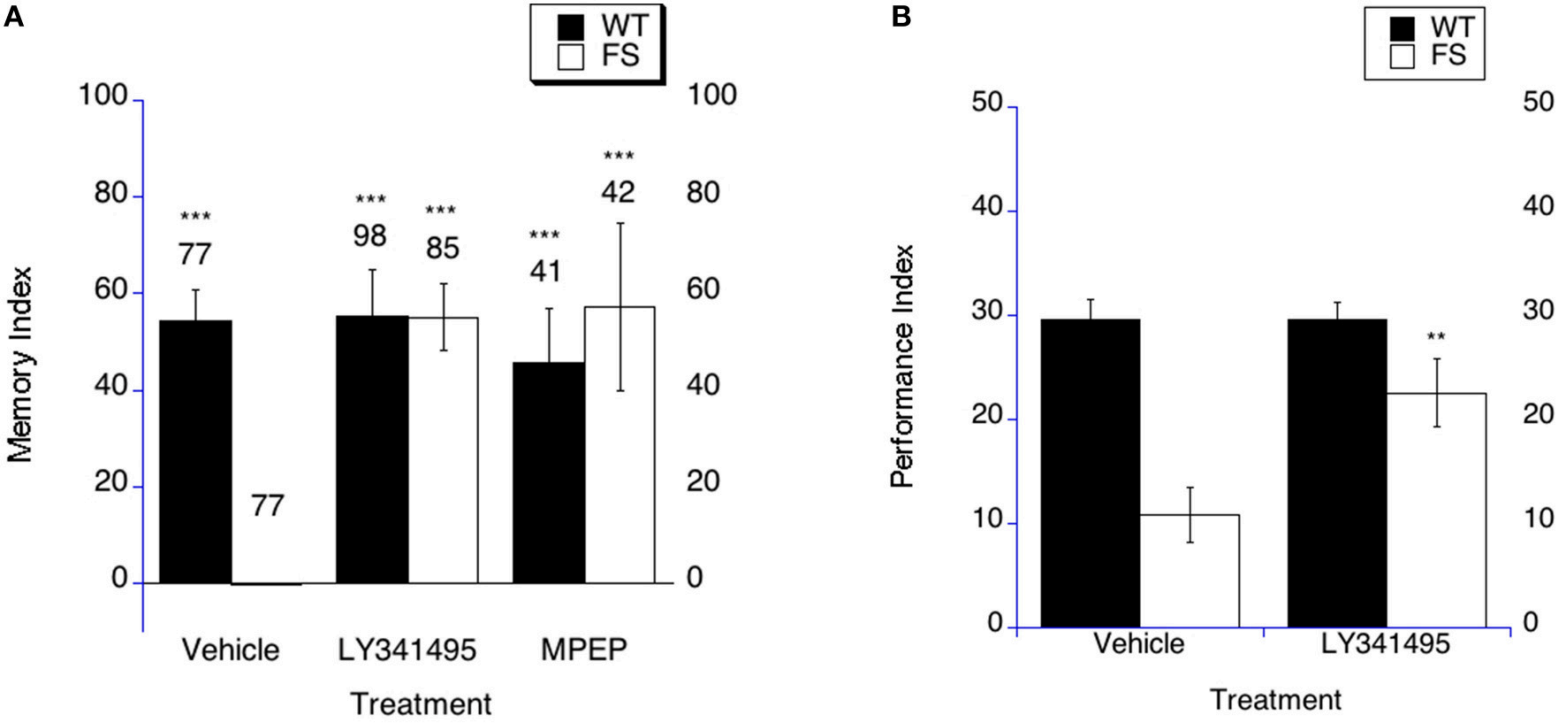
**Long-term memory is rescued by mGluR antagonist treatment in fragile X flies. (A)** Long-term memory (4 days post training) was measured in WT and FS flies that were administered vehicle control food or drug treatments contained in the food. Memory Index (±the error) was plotted; Ns are indicated above each bar for all groups. The levels of significance are ^*^p < 0.05; ^**^p < 0.01; ^***^p < 0.001, and are generated from comparisons of same genotype, same treatment between LTM trained and sham trained flies. Filled bars indicate WT males; open bars indicate FS males. Flies were raised on regular food and starting on the 1st day of adulthood placed on either vehicle control food containing DMSO vehicle or food supplemented with either 400 nM LY341495 or 8.6 μM MPEP. With vehicle treatment, FS males did not display long term memory. FS flies treated with LY341495 or MPEP did demonstrate long term memory. WT flies treated with either vehicle, MPEP or LY341495 demonstrated long term memory. **(B)** LY341495 rescues the olfactory-based long-term memory defect observed in *dfmr1* mutants. Flies were fed LY341495 or vehicle in the food for 9 days prior to spaced training. Long-term memory of olfactory conditioning after spaced training was significantly improved in FS mutants after being administered LY341495. The levels of significance are indicated as follows: ^**^p < 0.01; ^***^p < 0.001. No effect of treatment was observed in WT controls.

Previous studies have identified a mGluR antagonist treatment as a way to correct phenotypes for fragile X in the fly and mouse models as well as to correct symptoms in an exploratory analysis of 7 patients with full methylation of the promoter site in a small trial in patients (McBride et al., 2005; Dolen et al., 2007; Bolduc et al., 2008; Jacquemont et al., 2011; Michalon et al., 2012). In order to test the hypothesis that mGluR antagonists may rescue long-term memory in Fragile X flies, FS and WT flies were treated with 2-methyl-6-(phenylethynyl) pyridine (MPEP), LY341495 or the appropriate vehicle for 9 days starting on the day of eclosion as an adult. Flies then underwent sham or LTM training on day 10, and were subsequently placed back on MPEP, LY341495 or vehicle containing food for 3 days and tested for memory of training on day 4 post training. FS flies demonstrated rescued LTM at 4 days post training after treatment with MPEP or LY341495 (Figure 3A). In contrast, FS flies continued to have impaired LTM when treated with the vehicle control (Figure 3A). WT flies displayed intact LTM when treated with either of the mGluR antagonists or vehicle (Figure 3A).

In order to determine if mGluR antagonist treatment could rescue LTM in the olfactory-based LTM memory paradigm, we examined LTM in FS flies that were treated with LY341495 or vehicle. The FS flies on vehicle demonstrate worse LTM compared to the WT flies on vehicle treatment (Figure 3B, *p* < 0.001). FS flies that were treated with LY341495 displayed improved LTM compared to FS flies on vehicle treatment (Figure 3B as indicated by the asterisks). This represents a performance improvement in the FS flies, but not a full phenotypic reversal. WT flies demonstrated similar LTM on either LY341495 or vehicle treatment (Figure 3B).

### PDE-4 inhibitor treatment in adulthood rescues LTM in Fragile X flies

**Figure 4:**
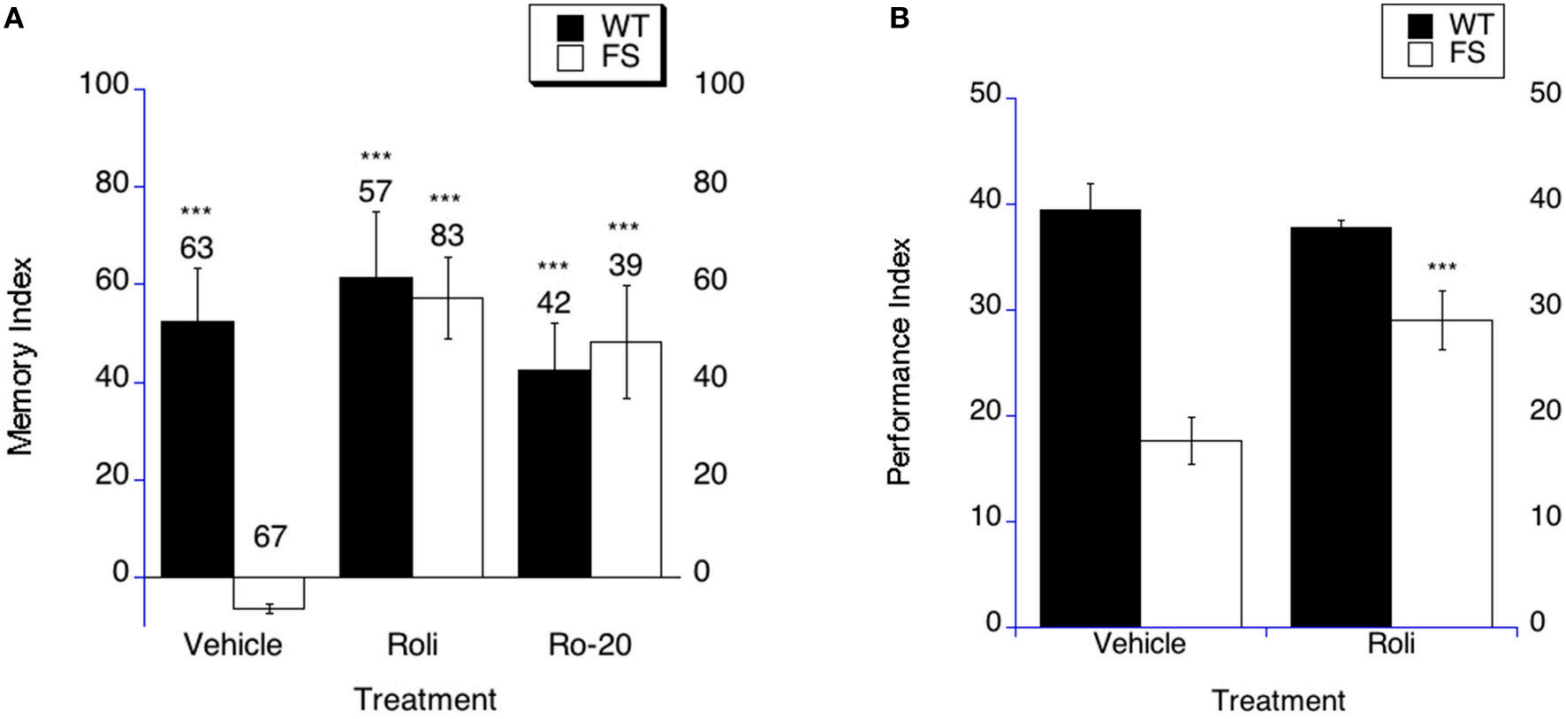
**Long-term memory is rescued by PDE-4 inhibitor treatment in fragile X flies. (A)** Long-term memory (4 days post training) was measured in WT and FS flies that were administered vehicle control food or drug treatments contained in the food. Memory Index (±the error) are plotted; Ns are indicated above each bar for all groups. The levels of significance are ^*^p < 0.05; ^**^p < 0.01; ^***^p < 0.001, and are generated from comparisons of same genotype, same treatment between LTM trained and sham trained flies. Filled bars indicate WT males; open bars indicate FS males. Flies were raised on regular food and starting on the 1st day of adulthood placed on either vehicle control food containing DMSO vehicle or food supplemented with either 50 μM Rolipram (Roli) or 20 uM Ro-20-1724 (Ro-20). With vehicle treatment, FS males did not display long-term memory. FS flies treated with rolipram or R0-20-1724 did demonstrate long-term memory. WT flies treated with either vehicle, rolipram or Ro-20-1724 demonstrated long-term memory. **(B)** Rolipram rescues the olfactory-based long-term memory defect observed in *dfmr1* mutants, but does not alter memory after massed training. Flies 1–3 days of age were fed Rolipram overnight before and after spaced training. Long-term memory of olfactory conditioning after spaced training was significantly improved in FS mutants after being administered rolipram. The levels of significance are indicated as follows: ^***^p < 0.001. No effect was observed in WT controls.

Previous studies have demonstrated efficacy of PDE-4 inhibition in rescuing phenotypes in the fly model and mouse model of fragile X (Choi et al., 2015). In order to test the hypothesis that PDE-4 inhibition may rescue LTM in Fragile X flies, FS and WT flies were treated with rolipram, Ro-20-1724 or the appropriate vehicle for 9 days starting on the day of eclosion as an adult. Flies then underwent sham or LTM training on day 10, and were subsequently placed back on rolipram, Ro-20-1724 or vehicle containing food for 3 days and tested for memory of training on day 4 post training. In mammals, both rolipram and Ro-20-1724 are widely used as PDE-4 inhibitors and both have been used in flies to inhibit PDE-4 at the concentrations used herein (Kanellopoulos et al., 2012; Choi et al., 2015). Additionally, the treatments have been shown to increase cAMP levels in flies (Kanellopoulos et al., 2012; Choi et al., 2015). FS flies demonstrated rescued LTM at 4 days post training after treatment with rolipram or Ro-20-1724 (Figure 4A). In contrast, FS flies continued to have impaired LTM when treated with the vehicle control (Figure 4A). WT flies displayed intact LTM when treated with either of the PDE-4 inhibitors or vehicle (Figure 4A).

In order to determine if PDE-4 inhibition could rescue LTM in the olfactory-based LTM memory paradigm, we examined LTM in FS flies that were treated with rolipram. We have previously shown that rolipram rescued short-term memory and did not affect olfaction or shock sensation (Choi et al., 2015). The FS flies on vehicle demonstrate worse LTM compared to the WT flies on vehicle treatment (Figure 4B, *p* < 0.0001). FS flies that were treated with rolipram displayed improved LTM compared to FS flies on vehicle treatment (Figure 4B as indicated by the asterisks). This represents a performance improvement in the FS flies, but not a full phenotypic reversal. WT flies demonstrated similar LTM on either rolipram or vehicle treatment (Figure 4B).

### The effects of chronic drug treatments that rescue mGluR-LTD on brain biochemistry

Previously it has been demonstrated that each of the three treatments used to rescue LTM in the fragile X fly model can also be used to rescue the phenotype of enhanced DHPG induced mGluR mediated LTD (long term depression) in the CA1 in the fragile X mouse model. It has been demonstrated that an 8 week treatment initiated in adulthood with either lithium, group II mGluR antagonist or PDE-4 inhibitor can normalize DHPG induced mGluR-LTD, but the effects of such prolonged treatments with these drugs under the conditions that demonstrated rescued DHPG induced mGluR-LTD has not been examined regarding brain biochemistry or lymphocyte biochemistry in fragile X mice (Choi et al., 2011, 2015). For the treatments with lithium or control chow or drug vs. vehicle, all mice were weighed 2–3 times weekly. For drug and vehicle injections, the injections were performed daily for 8 weeks starting at 8 weeks of age.

### The effect of chronic lithium treatment on GSK-3β phosphorylation

**Figure 5:**
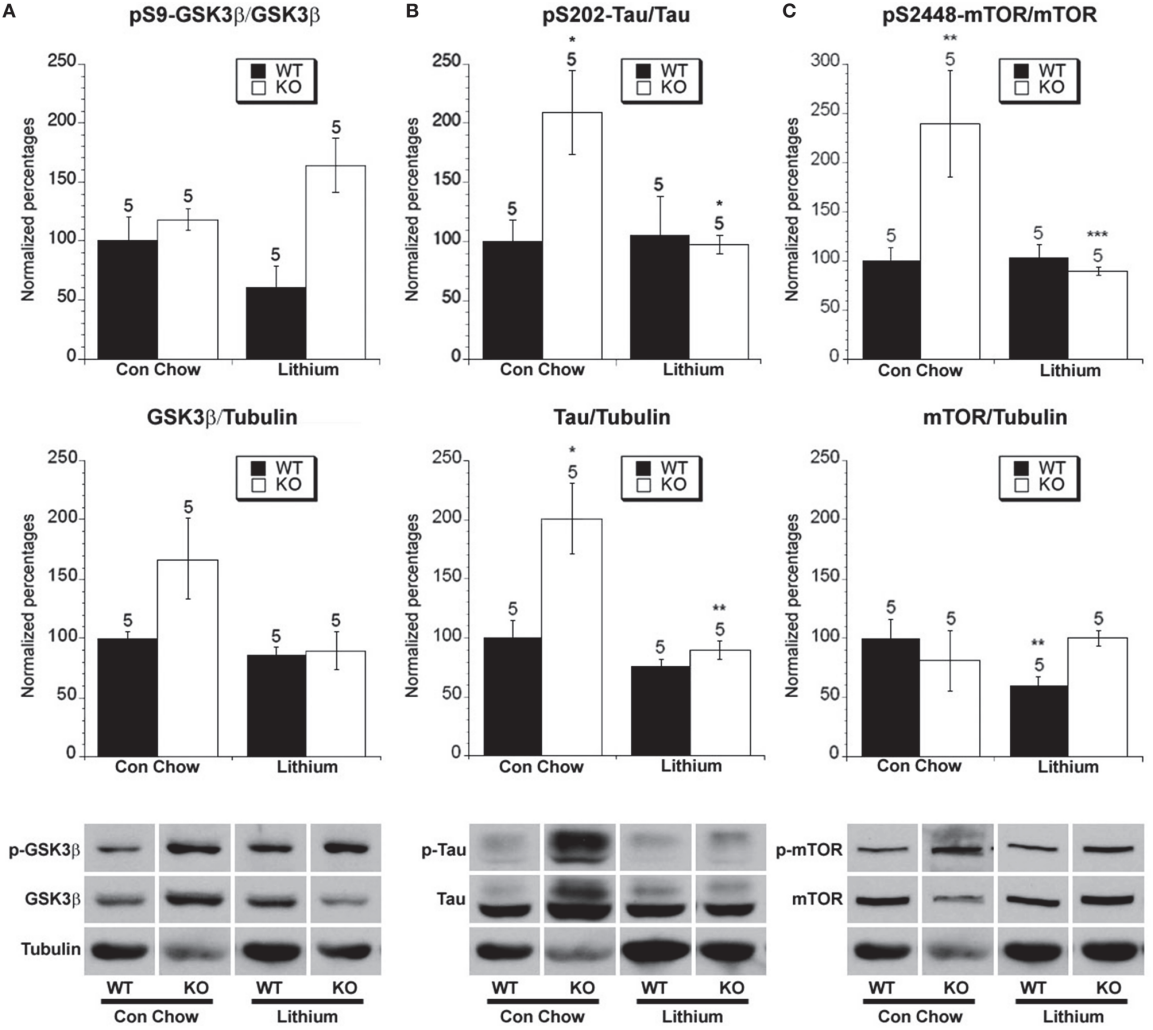
**The effect of lithium treatment on the phosphorylated and total levels of GSK-3β, tau and mTOR**. Eight week old *Fmr1* KO mice and age-matched WT mice were administered lithium-containing chow or control chow *ad libitum* until 5 months of age. All data in the top and middle panels are normalized to vehicle-treated WT mice. (^*^p < 0.05; ^**^ *p* < 0.01; ^***^ *p* < 0.001; n over each bar refers to number of mice). **(A)** Top Panel: There were no differences in the ratio of phosphorylated GSK-3β (Ser 9) to total GSK-3β in the hippocampus of *Fmr1* KO mice and WT mice after chronic lithium or control chow treatment. Middle Panel: There was no difference in the ratio of total GSK-3β to total α-tubulin. Bottom Panel: Representative bands of pS9 GSK-3β, total GSK-3β and α-tubulin. **(B)** Top Panel: There was an increased ratio of phosphorylated to total tau at S202 in *Fmr1* KO mice compared to WT mice on control chow. Lithium treatment reduced the ratio of phosphorylated to total tau in *Fmr1* KO mice compared to control treated *Fmr1* KO mice. Middle Panel: There was an increase in total tau protein in *Fmr1* KO mice compared to WT mice on control treatment. Lithium treatment decreased the total tau protein in *Fmr1* KO mice. Bottom Panel: Representative bands of pS202 tau, total tau and α-tubulin. **(C)** Top Panel: There was an increased ratio of phosphorylated to total mTOR at S2448 in *Fmr1* KO mice compared to WT mice on control chow. Lithium treatment reduced the ratio of phosphorylated to total mTOR in *Fmr1* KO mice compared to control treated *Fmr1* KO mice. Middle Panel: There was no difference in total mTOR protein in *Fmr1* KO mice compared to WT mice on control treatment. Lithium treatment decreased the total mTOR protein in WT mice. Bottom Panel: Representative bands of pS2448 mTOR, total mTOR and α-tubulin.

Lithium treatment inhibits GSK-3β activity by increasing phosphorylation at serine 9 on GSK-3β and competing with magnesium for the magnesium-binding site that is critically involved in GSK-3β activation (Klein and Melton, 1996; Grimes and Jope, 2001; Gurvich and Klein, 2002; Gould and Manji, 2005). Additionally, lithium inhibits Inositol-1-monophosphatase (IMPase) and Inositol polyphosphate 1-phosphatase (IPPase) activity, thereby reducing free InsP_3_ levels and lowering InsP_3_R mediated calcium signaling, which is also shown to be involved in LTD and more specifically mGluR-LTD in the hippocampus (Hallcher and Sherman, 1980; Baraban et al., 1989; Berridge et al., 1989; Worley et al., 1989; Berridge, 1993; Acharya et al., 1997, 1998; Fujii et al., 2000; Taufiq et al., 2005; Jo et al., 2008). GSK-3β is thought to be constitutively active unless it is inhibited by phosphorylation at serine 9. We did not find differences between WT control and *Fmr1* KO mice regarding alpha tubulin or beta actin levels, fitting with the previously published literature. It should be noted that in the representative traces there will appear to be more or less tubulin as less protein was added to some lanes to ensure that the proteins of interest that were being stained remained in the linear range and did represent an example of phosphorylated vs. total protein levels as shown on the graphs. There were no differences between WT and *Fmr1* KO mice on either control vehicle or lithium chow treatment in total GSK-3β protein levels (Figure 5A). There was not a significantly higher ratio of phosphorylated GSK-3β to total GSK-3β in WT mice compared to *Fmr1* KO mice on control vehicle chow treatment or lithium chow treatment (Figure 5A). However, there was a trend to lithium treatment increasing the ratio of phosphorylated GSK-3β to total GSK-3β in *Fmr1* KO, which has been demonstrated in some previous studies (Min et al., 2009b; Yuskaitis et al., 2010; Liu et al., 2011).

### The effect of treatment on GSK-3β mediated phosphorylation of tau

One of the most well characterized substrates of GSK-3β-mediated phosphorylation is the protein tau at serine 202, which was examined in order to get a more sensitive indication of GSK-3β activity (Dumont et al., 2011; Cavallini et al., 2013; Petry et al., 2014). There was increased total tau normalized to tubulin in *Fmr1* KO mice compared to WT mice on chronic control chow (Figure 5B). There was also an increased ratio of phosphorylated tau to total tau in *Fmr1* KO mice vs. WT mice on control chow treatment (Figure 5B). Therefore, *Fmr1* KO mice that are merely treated with control chow have increased levels of total tau and an increased percentage of tau phosphorylation at serine 202 compared to WT mice, indicating increased GSK-3β activity.

Lithium treatment lowered the amount of total tau in *Fmr1* KO mice compared to control chow treatment, returning it to near the level of WT mice on control chow. Lithium had no effect on total tau levels in WT mice (Figure 5B). In *Fmr1* KO mice, chronic lithium treatment significantly lowered the ratio of phosphorylated tau compared to control vehicle chow treatment (Figure 5B). This demonstrates that chronic lithium treatment reduces the activity of GSK-3β with regard to phosphorylation of the tau protein in *Fmr1* KO mice. Chronic lithium treatment did not lower the percentage of tau phosphorylation in WT mice. This result for WT mice does not fit all previous findings which have seen mixed effects on WT mice regarding tau phosphorylation after lithium treatment, in our study we have used an extended length of the treatment period compared to previous studies, thereby allowing a longer time for the WT mice to restore homeostatic balance of signaling (Hong et al., 1997; Noble et al., 2005; Engel et al., 2006).

Chronic lithium administration to FTDP-17 tau and GSK-3β overexpressing mice prevents tau hyperphosphorylation and neurofibrillary tangle formation, but pre-formed neurofibrillary tangles do not revert.

### The effect of lithium treatment on mTOR phosphorylation

Lithium treatment lowered the ratio of phosphorylated tau to the control chow treatment in *Fmr1* KO mice. These results raise the question of whether or not the increased phosphorylation of tau may result in altering the activity of proteins downstream of tau, which may be rescued by lithium treatment. It has been previously demonstrated that tau hyperphosphorylation can lead to increased mTOR activity in neurons (Khurana et al., 2006). Additionally, another potential consequence of upregulated mGluR signaling is upregulated mTOR activity since it has been demonstrated that DHPG-induced mGluR activation enhances mTOR activity, and that mTOR activity plays a role in DHPG-induced LTD (Hou et al., 2004; Banko et al., 2006; Figure 1A). Increased group I mGluR activity, through an unknown mechanism, results in increased GSK-3β activity in Fragile X mice (Min et al., 2009b). This could be through the coupling of group I mGluRs to Gi signaling, since even though they are predominantly coupled to Gq, they can also couple to a lesser degree to Gi in neurons (Kreibich et al., 2004). Group II mGluR activation should also increase GSK-3β activity through the activation of Gi signaling, leading to decreased cAMP signaling and decreased PKA activation, thereby relieving inhibition on GSK-3β signaling (Figure 1A). Since tau is hyperphosphorylated and it had been previously demonstrated that mTOR activity may be increased under certain conditions in *Fmr1* KO mice (Sharma et al., 2010), the phosphorylation status of mTOR at S2448, which stimulates activation, was investigated.

There was no difference in total mTOR protein in *Fmr1* KO mice compared to WT mice on chronic control vehicle chow. However, in WT, but not *Fmr1* KO mice, lithium treatment lowered the amount of total mTOR compared to control chow treatment, whereas no effect was observed in *Fmr1* KO mice (Figure 5C). There was an increased ratio of phosphorylated mTOR (s2448) to total mTOR in *Fmr1* KO mice vs. WT mice on control chow treatment (Figure 5C), fitting with some but not all of the previous literature (Osterweil et al., 2010; Sharma et al., 2010). Chronic lithium treatment did not lower the ratio of mTOR phosphorylation in WT mice, which fits with the finding that tau phosphorylation was not altered by lithium treatment in WT mice (Figure 5B). In *Fmr1* KO mice chronic lithium treatment significantly lowered the ratio of phosphorylated mTOR compared to control vehicle chow treatment (Figure 5C). This demonstrates that chronic lithium treatment reduces the activity of mTOR signaling in *Fmr1* KO mice and is consistent with lithium treatment lowering tau phosphorylation in *Fmr1* KO mice (Figure 5B).

### The effect of group II mGluR antagonist treatment on GSK-3β phosphorylation

**Figure 6:**
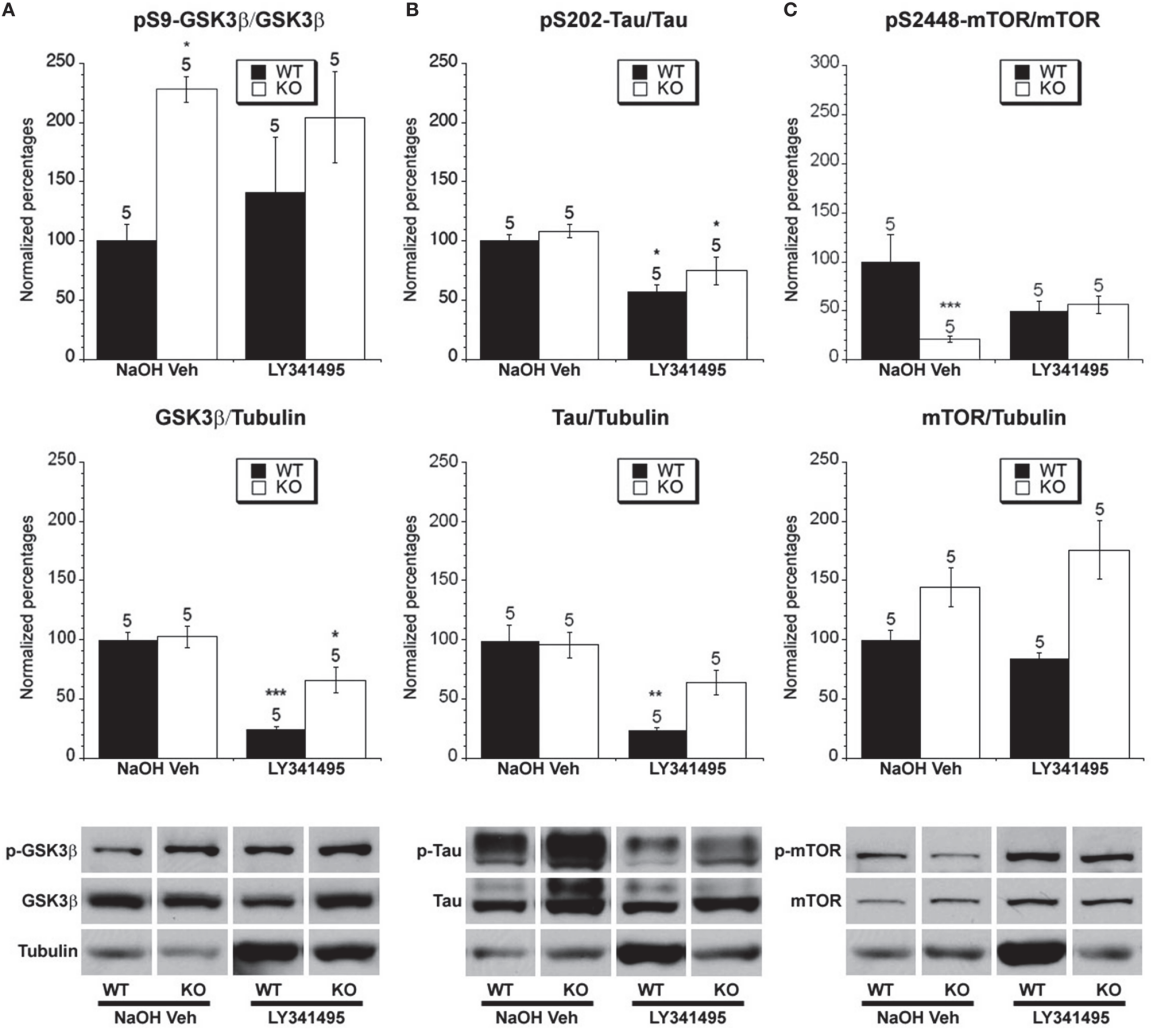
**The effect of LY341495 treatment on the phosphorylated and total levels of GSK-3β, tau and mTOR**. Eight week old *Fmr1* KO mice and age-matched WT mice were administered LY341495 or vehicle injections for 8 weeks. All data in the top and middle panels are normalized to vehicle-treated WT mice (^*^p < 0.05; ^**^ *p* < 0.01; ^***^ *p* < 0.001; *n* over each bar refers to number of mice). **(A)** Top Panel: There was an increased ratio of phosphorylated GSK-3β (Ser 9) to total GSK-3β in the hippocampus of *Fmr1* KO mice compared to WT mice receiving vehicle injection. LY341495 treatment did not alter the phosphorylation in *Fmr1* KO or WT mice. Middle Panel: There was no difference between the ratio of total GSK-3β to total α-tubulin in vehicle treated *Fmr1* KO and WT mice. LY341495 treatment decreased the levels of total GSK-3β in both *Fmr1* KO and WT compared to vehicle treated mice. Bottom Panel: Representative bands of pS9 GSK-3β, total GSK-3β and α-tubulin. **(B)** Top Panel: There was no difference in the ratio of phosphorylated to total tau at S202 in *Fmr1* KO mice compared to WT mice that were vehicle treated. LY341494 treatment reduced the ratio of phosphorylated to total tau in *Fmr1* KO and WT mice compared to vehicle treated mice. Middle Panel: There was no difference in total tau protein in *Fmr1* KO mice compared to WT mice on vehicle treatment. LY341495 treatment decreased the total tau protein WT mice. Bottom Panel: Representative bands of pS202 tau, total tau and α-tubulin. **(C)** Top Panel: There was a decreased ratio of phosphorylated to total mTOR at S2448 in *Fmr1* KO mice compared to WT mice on vehicle treatment. LY341495 treatment did not change the ratio of phosphorylated to total mTOR in *Fmr1* KO or WT mice compared to vehicle treated mice. Middle Panel: There was no difference in total mTOR protein in *Fmr1* KO mice compared to WT mice on vehicle treatment. LY341495 treatment did not change the total mTOR protein in *Fmr1* KO or WT mice. Bottom Panel: Representative bands of pS2448 mTOR, total mTOR and α-tubulin.

Antagonizing group II mGluRs is predicted to increase PKA activity after synaptic stimulation and thereby decrease GSK-3β activity (Fang et al., 2000; Li et al., 2000; Tanji et al., 2002; McBride et al., 2005). To determine if this is the case we examined the effect of LY341494 and vehicle treatment on WT and *Fmr1* KO mice. It should be kept in mind that this treatment involved injecting vehicle every day for 8 weeks which is very different than the lithium treatment and is thus not comparable to control chow treated groups, which were merely weighed several times weekly. In WT and *Fmr1* KO mice treated with vehicle no differences between total GSK-3β protein levels were observed, but LY341495 treatment decreased total GSK-3β protein levels in both WT and *Fmr1* KO mice compared to the levels of the vehicle treated mice (Figure 6A). Also, there was a significantly greater ratio of phosphorylated GSK-3β to total GSK-3β in the vehicle-treated *Fmr1* KO mice compared to vehicle-treated WT mice. Treatment with LY341495 had no effect on the GSK-3β ratio in either WT or *Fmr1* KO mice compared to vehicle treatment within the genotypes (Figure 6A). However, since the majority of GSK-3β is constitutively active looking at the phosphorylated ratio is not as sensitive as looking at a substrate that undergoes GSK-3β mediated phosphorylation, particularly in this case where the total level of GSK-3β protein was decreased by LY341495 treatment.

### The effect of group II mGluR antagonist treatment on GSK-3β mediated phosphorylation of tau

There were no differences in total tau protein levels between WT and *Fmr1* KO mice on chronic vehicle treatment (Figure 6B), indicating that daily handling for injection may have normalized total tau levels which could be related to findings showing effects on tau by different intensities of environmental enrichment (Billings et al., 2007; Hu et al., 2010, 2013). Chronic LY341495 treatment did reduce total tau levels in WT mice, but not in *Fmr1* KO mice. Furthermore, there was no difference in the ratio of phosphorylated to total tau between vehicle-treated WT mice and vehicle-treated *Fmr1* KO mice (Figure 6B). Chronic LY341495 treatment did decrease the percentage of tau phosphorylation at Ser-202 in both WT and *Fmr1* KO mice compared to vehicle-treated mice (Figure 6B). This suggests that chronic LY341495 treatment decreased GSK-3β activity in the WT and *Fmr1* KO mice as measured by examining the ratio of phosphorylated to total tau.

### The effect of group II mGluR antagonist treatment on mTOR phosphorylation

We next examined the effect of group II mGluR antagonist treatment on mTOR expression and activity levels. We did not find a difference in total mTOR between WT and *Fmr1* KO mice on chronic vehicle treatment or LY341495 treatment (Figure 6C). However, there was a lower ratio of phosphorylated to total mTOR between vehicle-treated *Fmr1* KO mice and vehicle-treated WT mice (Figure 6C). This result could indicate that handling of mice may act as a limited type of environmental enrichment, as was seen with tau phosphorylation. Chronic LY341495 treatment had no effect on the ratio of phosphorylated mTOR in the WT or *Fmr1* KO mice compared to vehicle treatment (Figure 6C).

### The effect of PDE4 inhibition on GSK-3β, tau, and mTOR phosphorylation levels

**Figure 7:**
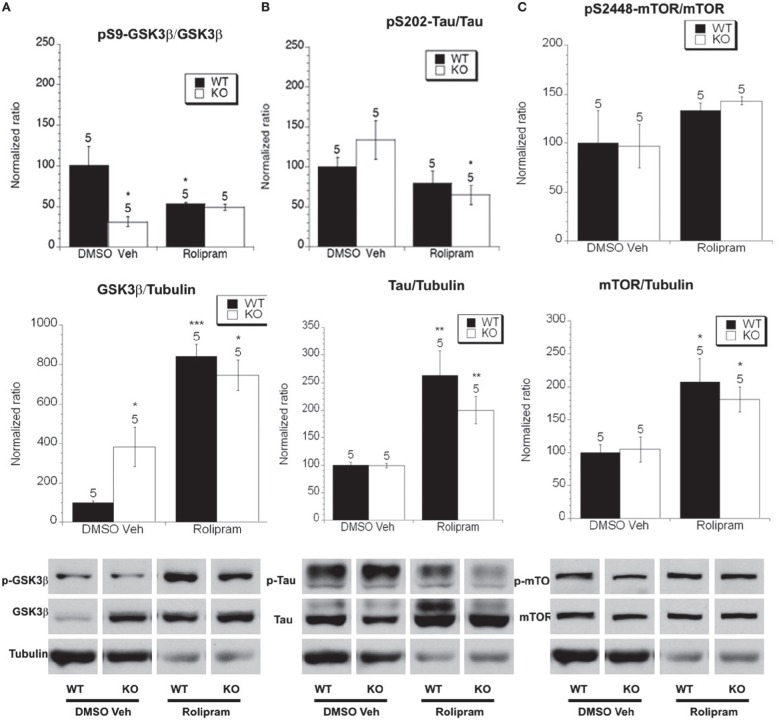
**The effect of rolipram treatment on the phosphorylated and total levels of GSK-3β, tau and mTOR**. Eight week old *Fmr1* KO mice and age-matched WT mice were administered rolipram or DMSO vehicle injections for 8 weeks. All data in the top and middle panels are normalized to vehicle-treated WT mice (^*^p < 0.05; ^**^p < 0.01; ^***^p < 0.001; *n* over each bar refers to number of mice). **(A)** Top Panel: There was an decreased ratio of phosphorylated GSK-3β (Ser 9) to total GSK-3β in the hippocampus of *Fmr1* KO mice compared to WT mice receiving vehicle injection. Rolipram treatment did not alter the phosphorylation in *Fmr1* KO and lowered it in WT mice. Middle Panel: There was an increased ratio of total GSK-3β to total α-tubulin in vehicle treated *Fmr1* KO compared to WT mice. Rolipram treatment increased the levels of total GSK-3β in both *Fmr1* KO and WT compared to vehicle treated mice. Bottom Panel: Representative bands of pS9 GSK-3β, total GSK-3β and α-tubulin. **(B)** Top Panel: There was no difference in the ratio of phosphorylated to total tau at S202 in *Fmr1* KO mice compared to WT mice that were vehicle treated. Rolipram treatment reduced the ratio of phosphorylated to total tau in *Fmr1* KO, but not in WT mice compared to vehicle treated mice. Middle Panel: There was no difference in total tau protein in *Fmr1* KO mice compared to WT mice on vehicle treatment. Rolipram treatment increased the total tau protein *Fmr1* KO and WT mice. Bottom Panel: Representative bands of pS202 tau, total tau and α-tubulin. **(C)** Top Panel: There was no difference in the ratio of phosphorylated to total mTOR at S2448 in *Fmr1* KO mice compared to WT mice on vehicle treatment. Rolipram treatment did not change the ratio of phosphorylated to total mTOR in *Fmr1* KO or WT mice compared to vehicle treated mice. Middle Panel: There was no difference in total mTOR protein in *Fmr1* KO mice compared to WT mice on vehicle treatment. Rolipram treatment increased the total mTOR protein in *Fmr1* KO and WT mice. Bottom Panel: Representative bands of pS2448 mTOR, total mTOR and α-tubulin.

We next examined the effect of rolipram treatment on *Fmr1* KO and WT mice. These sets of mice were also injected with drug or vehicle every day for 8 weeks. Total GSK-3β protein levels were higher in *Fmr1* KO mice on vehicle treatment than in WT mice. Additionally, in both *Fmr1* KO and WT mice rolipram increased total GSK-3Beta levels. There was a significantly lower ratio of phosphorylated GSK-3β to total GSK-3β in the vehicle-treated *Fmr1* KO mice compared to DMSO vehicle-treated WT mice. Treatment with rolipram had no effect on the GSK-3β ratio in either WT or *Fmr1* KO mice compared to vehicle treatment within the genotypes, although there was a trend toward increasing phosphorylation in the *Fmr1* KO mice (Figure 7A).

We next examined total and phosphorylated levels of tau and found that there were no differences in total tau protein levels between WT and *Fmr1* KO mice on chronic DMSO vehicle treatment (Figure 7B), however rolipram treatment increased total tau in both WT and *Fmr1* KO mice. There was no difference in the ratio of phosphorylated to total tau between vehicle-treated WT mice and *Fmr1* KO mice (Figure 7B). Chronic rolipram treatment did however decrease tau phosphorylation levels in *Fmr1* KO mice vs. vehicle treated *Fmr1* KO mice (Figure 7B). This suggests that chronic rolipram treatment decreased GSK-3β activity in the *Fmr1* KO mice as measured by examining the ratio of phosphorylated tau to total tau.

Finally, we examined total and phosphorylated levels of mTOR. We did not detect a difference in total mTOR between vehicle treated WT and *Fmr1* KO mice but rolipram treatment increased total mTOR levels in both WT and *Fmr1* KO mice (Figure 7C). There was no difference in the ratio of phosphorylated to total mTOR between vehicle-treated *Fmr1* KO and WT mice and, chronic rolipram treatment had no effect on the ratio of phosphorylated mTOR in the WT or *Fmr1* KO mice compared to vehicle treatment (Figure 7C).

### The effect of modulating cAMP signaling in bioassay of ERK activation in lymphocytes

**Figure 8:**
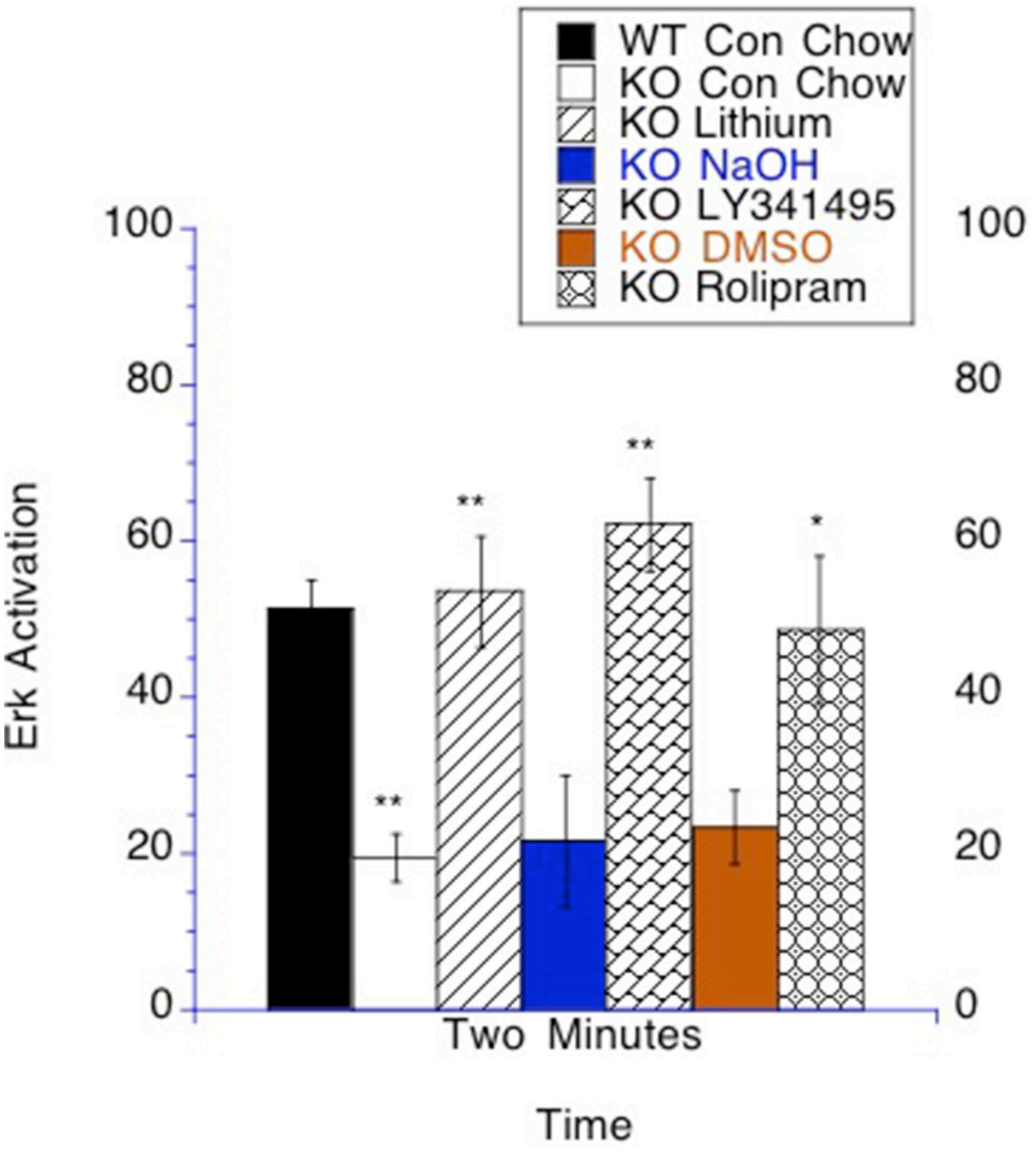
**Bioassay**. ERK activation by PMA stimulation is altered in lymphocytes by lithium, LY341495 or rolipram treatment in *Fmr1* KO mice. The number of T lymphocytes per field was set to count the same number of cells for each analyzed sample. At 2 min after stimulation there was more p-ERK staining in WT mice compared to *Fmr1* KO on control chow. The time point of 2 min post stimulation is graphed with the unstimulated value subtracted from the stimulated sample. *Fmr1* KO mice on lithium chow treatment had increased p-ERK compared to *Fmr1* KO mice placed on control chow. *Fmr1* KO mice on LY341495 treatment had increased p-ERK compared to *Fmr1* KO mice on vehicle treatment. *Fmr1* KO mice on rolipram treatment had increased p-ERK compared to *Fmr1* KO mice on DMSO vehicle treatment ^*^p < 0.05 and ^**^p < 0.01.

There is an interest in developing bioassays in the fragile X field that could potentially be minimally invasive and used in the clinic (Berry-Kravis et al., 2008; Weng et al., 2008; Erickson et al., 2011b; Hoeffer et al., 2012). By collecting blood samples and isolating lymphocytes, the time course of ERK activation in lymphocytes can be measured after phorbol-12-myristate-13-acetate (PMA) treatment (Chow et al., 2001; Tong et al., 2006), which has been utilized as a bioassay in fragile X mice and patients (Berry-Kravis et al., 2008; Weng et al., 2008; Erickson et al., 2011b). A major question was whether these three treatments, which all increase cAMP signaling and decrease GSK-3β mediated tau phosphorylation, would have measurable effects in the signaling measured in this bioassay. It has previously been shown that p-ERK activation after PMA stimulation is slower in fragile X mice and patients and that this deficit can be corrected by lithium treatment (Berry-Kravis et al., 2008). We found that at the time point of 2 min after ERK activation by PMA there was a difference between control and *Fmr1* KO mice on control chow. In mice on control chow, *Fmr1* KO mice had decreased p-ERK compared to WT mice, which is consistent with the previous literature (Berry-Kravis et al., 2008; Weng et al., 2008). Fitting with the previous literature, lithium treatment normalized p-ERK levels to WT levels (Berry-Kravis et al., 2008; Weng et al., 2008; Figure 8). Additionally, both LY341495 and rolipram treatment normalized p-ERK levels at 2 min post PMA stimulation. In contrast, the vehicle treatments for each had no measureable effect on ERK activation (Figure 8).

## Discussion

### Social interaction

Treatment with lithium, mGluR antagonists and PDE-4 inhibitors given in adulthood was able to increase social interaction, which is normally suppressed in the fragile X flies. However, treatment of the control flies with lithium and mGluR antagonists decreased social interaction. This finding fits with previous work demonstrating that a heterozygous loss of function mutation in DmGluRA can suppress social interaction in flies (Schoenfeld et al., 2013).

### The effects of modulating cAMP signaling on LTM in the *dfmr1* mutants

In our initial studies of the Fragile X fly model we found that lithium treatment given in development or adulthood rescues social interaction, immediate recall and short-term memory (McBride et al., 2005). Herein we have extended these findings to include LTM. We demonstrate that treatment started in adulthood is able to rescue LTM in the fly model of fragile X. Since our initial studies with the *Drosophila* model, it has now been demonstrated that lithium treatment can rescue the several phenotypes in *Fmr1* KO mice (Min et al., 2009b; Mines et al., 2010; Choi et al., 2011; Liu et al., 2011, 2012; Melancon et al., 2012; Hashimoto et al., 2013). Moreover in a recent open label trial it has been shown that lithium can positively affect some behaviors in Fragile X patients (Berry-Kravis et al., 2008).

Herein we have also found that treatment with mGluR antagonists in adulthood can rescue LTM in the fly model of fragile X. Previously it has been demonstrated that pharmacologic treatment with mGluR antagonists in adulthood alone could rescue social interaction, immediate recall and short-term in the fly model of Fragile X (McBride et al., 2005; Bolduc et al., 2008; Choi et al., 2010). Additionally, treatment for 8 weeks in adulthood was able to normalize the mGluR dependent LTD phenotype in *Fmr1* KO mice (Choi et al., 2011).

Previous research has also shown that pharmacologic treatment with PDE-4 inhibitors beginning in adulthood could rescue social interaction, immediate recall and short-term in the fly model of Fragile X (Choi et al., 2015). Additionally, treatment for 8 weeks in adulthood was able to normalize the mGluR dependent LTD phenotype in *Fmr1* KO mice (Choi et al., 2015). Herein we demonstrate that treatment with PDE-4 inhibitors in adulthood was able to rescue LTM in two paradigms for LTM in the fly model of fragile X.

### The effects of modulating cAMP signaling in the *Fmr1* KO mice

To determine the impact of modulating cAMP levels on signaling deficits in the Fragile X models, we turned to the mouse model. This is because of the ability to dissect more homogenous brain tissue and the readily available and validated antibodies. We examined hippocampal signaling via western blot of mice chronically treated (for 8 weeks in adulthood) with three distinct strategies that have been shown to rescue DHPG induced mGluR dependent LTD, which are lithium, LY341495 and rolipram (Choi et al., 2011, 2015). We chose to focus on 3 potentially critical nodes of signaling, GSK-3β, tau, and mTOR. We did not find consistent changes in the ratio of phosphorylated to total GSK-3β across the drug treatments. However, we examined the phosphorylation of tau by GSK-3β as a surrogate marker for GSK-3β activity and found that all three treatments were able to reduce the ratio of phosphorylated to total tau.

It is interesting to note that tau hyperphosphorylation is thought to play a role in two common neurodegenerative diseases, Alzheimer's disease and frontotemporal dementia. This is an interesting similarity as we have already found a strong genetic interaction between *Drosophila dfmr1* and *presenilin* (*psn*) genes. *Psn* is the *Drosophila* homolog of the vertebrate *presenilin* genes (*PS1* and *PS2*). In humans, mutation of *PS1* or *PS2* leads to familial Alzheimer's disease (Small and Gandy, 2006). We previously reported that the *psn* and *dfmr1* genes genetically interact in a transheterozygous context to severely affect social interaction in the courtship assay (McBride et al., 2010). In studies by others, the amyloid precursor protein (APP) mRNA has been shown to bind FMRP (Darnell et al., 2001, 2011), APP protein has been demonstrated to be upregulated in the fragile X mouse (D'agata et al., 2002) and differential processing of the fragments of ABeta 1-40 and ABeta 1-42 have been demonstrated to occur in a similar manner as those linked to some other presenilin FAD mutations (Westmark et al., 2011). Hyperphosphorylation of tau is thought to be a critical event in the progression of both Alzheimer's disease and frontotemporal dementia (Small and Gandy, 2006).

In our biochemical studies, we did not find a common effect from our drug studies on the ratio of phosphorylated to total mTOR. Lithium treatment given for 8 weeks in adulthood was able to reduce mTOR phosphorylation, but treatment with LY341495 and rolipram did not reduce mTOR phosphorylation. This leaves open the question of how critical mTOR activity is to the rescue of phenotypes in the fragile X model for exploration in future studies. Since cAMP increases could potentially mediate mTOR inhibition through increasing the activity of LKB1 to activate AMPK or through PKA without requiring AMPK activity changes, which may not result in changes in mTOR phosphorylation, this highlights the need to look at proteins downstream of mTOR activity as a potentially more accurate marker of mTOR activity (Xie et al., 2011; Laplante and Sabatini, 2012).

### Clinical prospects

The three strategies to increase cAMP signaling all normalize p-ERK levels (as a marker of activation) after PMA stimulation in lymphocytes in the *Fmr1* KO mice. These findings add to a growing body of evidence that using lymphocytes as surrogate markers for a bioassay of fragile X may be useful in clinical studies (Berry-Kravis et al., 2008; Weng et al., 2008; Erickson et al., 2011a; Hoeffer et al., 2012). Lithium is currently available for other indications clinically and indeed a small trial already yielded some positive results in Fragile X patients (Berry-Kravis et al., 2008). Two PDE-4 inhibitors are currently available clinically, apremilast approved for psoriatic arthritis and roflumilast approved for the treatment of chronic obstructive pulmonary disease. Both have poor blood brain barrier penetration (Kavanaugh et al., 2015; Martinez et al., 2015), although roflumilast has been demonstrated to have enough blood brain barrier penetration to alter neuronal signaling (Jabaris et al., 2015). Furthermore, resveratrol is a natural compound found in red wine that has been studied regarding anti-aging properties and in the context of Alzheimer's disease. Resveratrol crosses the blood brain barrier and has been shown to be able to inhibit PDE4 activity and increase AMPK activity which is downstream of PKA (Chung, 2012; Park et al., 2012; Bitterman and Chung, 2015; Pasinetti et al., 2015). Neither PDE-4 inhibitors, nor resveratrol has been studied in a clinical trial involving Fragile X patients.

Currently, the only group II mGluR antagonist in Phase 1 clinical trials is BCI-838, for which the majority of preclinical research has had a focus on depression and Alzheimer's disease (Hashimoto et al., 2013; Kim et al., 2014). In the fly there is just one functional mGluR receptor which signals through both Gi and Gq (Bogdanik et al., 2004). mGluR antagonist treatment inhibits the Gi coupled signaling increasing cAMP levels after synaptic stimulation activating PKA which inhibits GSK-3β and thereby increase cAMP signaling (Figure 1A; Bogdanik et al., 2004; McBride et al., 2005; Kelley et al., 2007; Kanellopoulos et al., 2012; Choi et al., 2015). This work adds to the current literature in favor of clinical trials for each of these potential treatments in fragile X.

## Materials and methods

### Drosophila strains and drug testing

The *Drosophila* strains were cultured as in Dockendorff et al. (2002), McBride et al. (2005), and Banerjee et al. (2010). For flies tested in the courtship studies are from Banerjee et al. (2010), so that the eye color is slightly more red to enhance naive courtship equally in the FS and WT flies, these lines were derived from the original FS and WT lines from Dockendorff et al. (2002). Drugs were obtained from Tocris-Cookson (UK), solubilized according to manufacturer's instructions and mixed into solid fly food at the appropriate concentration. Vehicle for each drug was added to the appropriate control food for each experiment. For the lithium treatment courtship experiments we used 5 mM LiCl or a 5 mM NaCl vehicle treatment. For the mGluR antagonist courtship experiments we used 8.6 μM MPEP or 400 nM LY341495 or a DMSO vehicle treatment. For the PDE-4 inhibitor courtship experiments we used 50 μM rolipram, and 20 μM Ro-20-1724 in the experiments presented with a DMSO vehicle. For the courtship studies flies were placed on the food on the day of eclosion until the night before the training session and then placed back on drug containing food until the night before testing. For olfactory classical conditioning, we used 50 μM rolipram with 5% sucrose and 5% DMSO on a filter paper. Flies were fed overnight after having been starved for 3 h. The flies were trained and tested the next morning. The control consisted of 5% sucrose and 5% DMSO. For lithium (5 mM) and LY341495 (600 nM), 1–3 days old flies were placed on drug/vehicle containing food for 9 days prior to space training.

### Behavioral training and testing in the conditioned courtship paradigm

Virgin male flies were collected under anesthesia and all testing was performed as previously described (McBride et al., 1999, 2005; Choi et al., 2010). Male flies were collected and placed on either vehicle or drug treatment for 10 days after eclosion. Virgin XX, yf females were collected on the day of eclosion and kept in food vials. Flies were aged in a 12:12 LD before behavioral training and testing was performed during the relative light phase. All male subjects were transferred to fresh control food the day before testing and assigned to random groups for blinded behavioral training and testing. All training and testing was performed blind to genotype and treatment. A courtship (CI) was calculated following testing as the percentage of total observation time spent courting (Siegel and Hall, 1979; McBride et al., 2005). A memory index was calculated as CI naive-CI trained/CI naive × 100, the error calculated for the memory index was the average of the standard errors of the naive and trained groups scaled to the calculated memory index, keeping the percentage of error the same (Keleman et al., 2007). Sensory and motor testing for these treatments has been published on previously for these flies (McBride et al., 2005; Choi et al., 2010, 2015).

### Courtship-based long-term memory

Long-term memory training was performed in flies that were 10 days post eclosion and then testing was performed on the fourth day post training as described previously (McBride et al., 1999; Banerjee et al., 2010). Statistical analyses was performed as described previously with courtship indices (CIs) of tested males were subjected to arcsin square root transformations to approximate normal distributions (McBride et al., 1999, 2005). ANOVAs and unpaired student *t*-tests were performed on planned comparisons of arcsin square root transformed data with the *post-hoc* analysis used for the comparisons being the Bonferroni analysis. Since some of the set of data could not be proven to be normal we proceeded with performing non parametric Mann–Whitney U test on all of the planned comparisons (naive vs. trained) (Siegel, 1957; Keleman et al., 2007) to generate the critical *p*-values that are shown in the figures (McBride et al., 1999, 2005; Banerjee et al., 2010; Schoenfeld et al., 2013). For all of the comparisons there was agreement between the statistical tests either using the ANOVAs with Bonferroni analysis or the Mann-Whitney U test as to whether or not the comparison achieved significance. All statistics were performed using Statview 3.0 and Prism 5.0.

### Pavlovian olfactory learning and memory

*Drosophila* were raised at 22°C and placed at 25°C overnight prior to behavioral experiments. Adult *Drosophila* 3–5 days old were trained and tested with the classical conditioning procedure of Tully and Quinn (1985). Flies were treated with either drug or vehicle contained in the food for 1 day prior to testing. About 100 flies were trapped inside a training chamber, covered with an electrifiable copper grid. Flies were allowed 90 s to acclimate and then were exposed sequentially to two odors, 3-octanol (OCT) and 4-methylcyclohexanol (MCH), carried through the chamber in a current of air (750 mL/min; relative concentrations of OCT and MCH were adjusted so that naive flies distributed themselves 50:50 in the T-maze). Flies first were exposed for 60 s to the conditioned stimulus (CS+; either OCT or MCH), during which time they received the unconditioned stimulus (US; twelve 1.25 s pulses of 60V DC electric shock at 5 s interpulse intervals). After the CS+ presentation, the chamber was flushed with fresh air for 45 s. Then flies were exposed for 60 s to a second, control stimulus (CS-; either MCH or OCT depending on the odor the flies were shocked to in the first step), which was not paired with electric shock. After the CS-presentation, the chamber was again flushed with fresh air for 45 s. The training was repeated for 10 times with 15 min rest interval for memory after spaced training. For training of massed training, 10 training sessions are provided but without rest interval. At 1 day after training, flies are inserted in the t-maze. To test for conditioned odor avoidance after classical conditioning, flies were tapped gently from the training chamber into an elevator-like compartment that transports them to the choice point of the T-maze. Ninety seconds later, the flies were exposed to two converging current of air one carrying OCT, the other MCH, from opposite arms of the T-maze. Flies were allowed to choose between the CS+ and CS− for 120 s, at which time they were trapped inside their respective arms of the T-maze (by sliding the elevator out of register), anesthetized and counted. Two groups of flies were trained and tested in one complete experiment. The CS+ was OCT and the CS− was MCH for one group; the CS+ was MCH and the CS− was OCT for the second group.

#### Drug feeding

For acute feeding, flies were starved for 4 h and then place in a vial containing a filter paper with 200 μL of solution. The vehicle contained 5% sucrose with DMSO 5% for Rolipram. Flies were exposed to 50 μM for the treatment.

#### Analysis of the olfactory memory performance

The performance index (PI) was calculated as the average of the fraction of the population avoiding the shock-associated odor minus the fraction avoiding the control odor for each group of flies trained in one experiment. In other words, the PI enumerates the distribution of flies in the T-maze as a normalized “percent correctly avoiding the shock-paired odor” and ranges from 0 for a 50:50 distribution to 100 for a 100:0 distribution. Data from an experiment were subjected to a one-way ANOVA (JMP from SAS, Inc.), followed by planned pairwise comparisons as indicated in text and figure legend. An α = 0.05 was corrected for multiple comparisons using Bonferroni. Post-test analysis was performed with Tukey test. Comparisons with one, two, or three asterisks indicate significances of *P* < 0.05, 0.001, and 0.0001, respectively. All graphs depict mean ± s.e.m.

### Mouse studies

All animal studies were conducted in accordance with protocols approved by the Institutional Animal Care and Use Committee at the Drexel University College of Medicine and Albert Einstein College of Medicine. Mice that are homozygous (female) or hemizygous (male) for a targeted mutation in the *Fmr1* gene on the X chromosome and backcrossed for 11 generations onto the FVB background were purchased from the Jackson Laboratory (Bar Harbor, ME) and bred in-house (*Fmr1* KO mice, stock number 004624; Bakker et al., 1994). Mice of the appropriate control strain were also purchased from Jackson and bred in-house (stock number 004828). To verify the genotype of the animals, DNA was extracted from tail tissue and the following primers were used for PCR analysis: mutant primers 5′-CCg gTT CTT TTT gTC AAg ACC g-3′ and 5′-Cgg CAg gAg CAA ggT gAg AT-3′; wild-type primers 5′-gTg gTT AgC TAA AgT gAg gAT gAT-3′ and 5′-CAg gTT TgT Tgg gAT TAA CAg ATC-3′. All mice were subjected to a 12 h:12 h light: dark cycle. Food and water were provided *ad libitum*. Only male mice were used for experimentation in this paper.

### Drug administration

Drug treatments were performed using the same treatment protocols utilized in Choi et al. (2011) and Choi et al. (2015). R, S-DHPG and LY341495 were purchased from Tocris (St. Louis, MO). DHPG was solubilized as a 50 mM stock in water; LY341495 was prepared as 100 mM stock in equimolar NaOH as recommended by the company. Solubilized drugs were aliquoted and frozen at −20°C. Fresh stocks were prepared weekly for DHPG, and biweekly for LY341495 and NaOH vehicle.

#### LY341495

Eight week old male *Fmr1* KO mice and age-matched male control mice were administered 0.9% saline containing 0.3 mg/kg LY341495 or vehicle alone via subcutaneous injections once daily for 8 weeks. This dose has previously been to shown to be the lowest dose that reverses the *in vivo* effects of a group II mGluR agonist (Ornstein et al., 1998). The mice were given at least a 2-week drug and handling hiatus prior to testing. Mice were weighed weekly to help monitor general well-being.

#### Lithium

Eight week old male *Fmr1* KO mice and age-matched male WT control mice were administered custom-made chow containing 2.4 g/kg lithium carbonate or vehicle (Bio-Serv, Frenchtown, NJ) for 8 weeks. This dose and route of administration have previously been shown to achieve clinically relevant concentrations of serum lithium (0.6–1.2 mM) in mice (Son et al., 2003; Su et al., 2004; Choi et al., 2011). Food and water were provided *ad-libitum*. Serum lithium concentration was determined by collecting trunk blood, isolating the serum by centrifugation (11,000 rpm, 5 min) and then utilizing services provided by the Hospital of the University of Pennsylvania. The tested samples of trunk blood demonstrated that the serum lithium concentration was in the clinically relevant range for all treated mice and none of the control mice. Mice were weighed 2 to 3 times weekly to help monitor general well-being.

### Western blotting

Mice were deeply anesthetized using isoflurane and decapitated. Hippocampi were immediately dissected on ice and placed in ice-cold homogenization buffer (in mM: HEPES, 5; MgCl_2_, 1; EGTA, 2; sucrose, 0.32; protease and phosphatase inhibitors). The tissue was homogenized in a 7mL glass homogenization tube using 25 strokes, transferred to new tubes and sonicated; the temperature was maintained at 4°C throughout all procedures. Protein assay was performed on the samples before storing them at −80°C.

For Western blotting, 30–60 μg of protein from the hippocampal lysates were separated by SDS-PAGE and transferred to nitrocellulose membranes, which were blocked (5% milk in 1x TBS and 0.01% Tween-20) then probed with primary followed by secondary antibodies. For sequential blotting, the membranes were stripped (Restore Western Blot Stripping Buffer, Pierce, IL), washed (1X TBS with 0.01% Tween-20) then reprobed with primary and secondary antibodies. All membranes were developed using enhanced chemiluminescent detection (Amersham, NJ). Western blots were analyzed using ImageJ software (NIH, MD) for densitometric analysis and normalized to the quantity of α-tubulin and β-actin.

Antibodies against GSK-3β (total and phospho Ser-9), mTOR (total and phospho Ser-2448), β-actin and α-tubulin were purchased from Cell Signaling (Danvers, MA). Antibodies against tau (DP9 against total tau and CP13 against phospho Ser-202) were generous gifts from Dr. Peter Davies (Albert Einstein College of Medicine). Statistical analysis was performed as described previously (Sharma et al., 2010) with arcsin square root transformations to approximate normal distributions (McBride et al., 1999, 2005) and one way ANOVAs were performed on planned comparisons of arcsin square root transformed data with the *post-hoc* analysis used for the comparisons being the Bonferroni analysis.

#### ERK activation assay

ERK activation assay was performed as described in Weng et al. (2008) and Berry-Kravis et al. (2008). AlexaFluor 647 was used for staining of p-ERK ½ (MAPK42/44) (purchased from Cell Signaling). For these studies multiple time points after PMA stimulation were measured by flow cytometry, only the 2 min time point minus the 0 min unstimulated time point are graphed. Statistical analysis was performed by the Mann-Whitney test on preplanned comparisons.

## Author contributions

All authors listed, have made substantial, direct and intellectual contribution to the work, and approved it for publication.

### Conflict of interest statement

The authors declare that the research was conducted in the absence of any commercial or financial relationships that could be construed as a potential conflict of interest.
